# Challenges for the effectiveness of infectious disease interventions during global change

**DOI:** 10.1038/s44528-026-00044-z

**Published:** 2026-10-07

**Authors:** Justin Sheen, Emily Howerton, Holly A. Burrows, Benjamin L. Rice, Chadi M. Saad-Roy

**Affiliations:** 1 Department of Global Health and Population, Harvard T.H. Chan School of Public Health, Boston, MA USA; 2 Department of Ecology and Evolutionary Biology, Princeton University, Princeton, NJ USA; 3 Department of Mathematics, University of British Columbia, Vancouver, BC Canada; 4 Department of Microbiology and Immunology, University of British Columbia, Vancouver, BC Canada; 5 Biodiversity Research Centre, University of British Columbia, Vancouver, BC Canada

**Keywords:** Diseases, Ecology, Ecology

## Abstract

Global change is expected to have complex effects on pathogen biology, yet how it will affect interventions has received less attention. Here, we explore how global change challenges the ability of interventions to act as ecological suppressors of transmission.

## Introduction

Global change, including climate change, land-use change, and increased human mobility, is expected to have complex effects on the transmission, evolution, and emergence of infectious diseases^1, 2^. Current research agendas aim to understand how these environmental and anthropogenic changes will affect pathogen transmission to anticipate effects on infection burden^3–7^. However, the impacts of global change on interventions for disease control is a key dimension that has been studied in comparatively less detail^8–11^. Many pathogens are kept under control by the continuous efforts of public health interventions in both human and animal populations; understanding how the effectiveness of these interventions may change in the future is essential for continued mitigation of transmission.

One explanation for comparatively less focus on global change and public health interventions is the historical separation between the disciplines of infectious disease ecology and public health^12, 13^. However, interventions act as ecological suppressors in dynamical disease systems, as they reduce contact, infectiousness, and susceptibility, constraining the basic reproduction number of pathogens, ${{{\mathcal{R}}}}_{0}$, or the number of secondary infections from a single infectious individual in a completely susceptible population, thereby preventing epidemic growth^14^. The challenges that global change pose to the protective effectiveness of these ecological suppressors present a timely opportunity for the integration of ecological frameworks within public health intervention approaches. A synthesis of these two perspectives can guide efficient and optimal interventions against endemic and emerging pathogens in the face of global change.

In this Comment, we describe how common disease control interventions are susceptible to accelerating global change through altered intervention effectiveness, implementation disruptions, and complications that emerge from novel transmission pathways. We explore where improvement of interventions to protect against these susceptibilities is most urgently needed. We then identify ways to mitigate risks of global change in order to create more robust control strategies.

## Vulnerability of interventions to global change

Interventions are ecological suppressors, meaning they constrain the basic reproduction number of pathogens, ${{{\mathcal{R}}}}_{0}$^14^. In the simplest model of transmission, ${{{\mathcal{R}}}}_{0}$ is defined as:

$${{{\mathcal{R}}}}_{0}=\dfrac{{c}{\cdot} {p}}{\gamma }$$

**Fig. 1:**
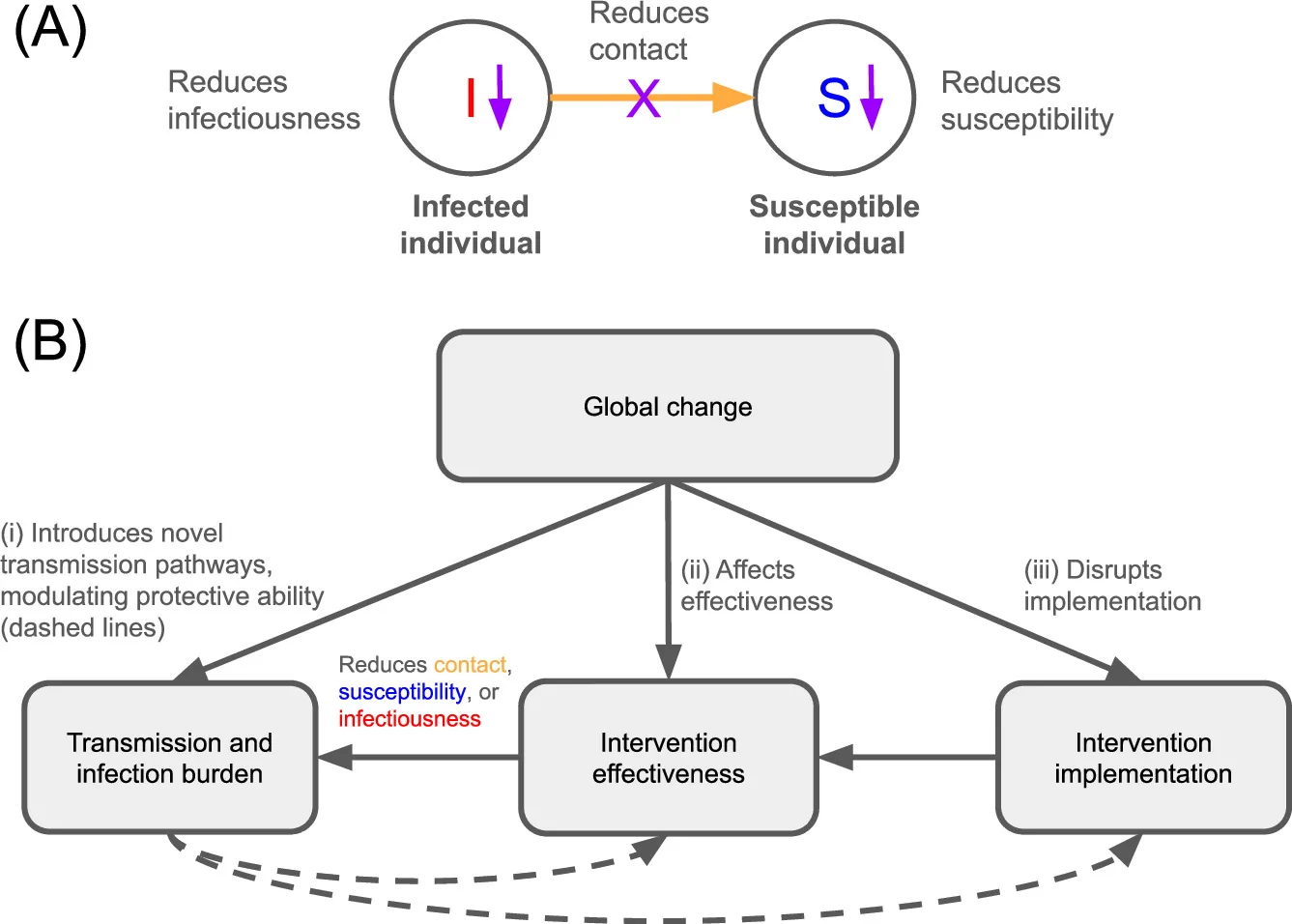
How global change affects infection burden and disease control interventions. **A** Interventions act as ecological suppressors (in purple) that reduce contact (*c* in the ${{{\mathcal{R}}}}_{0}$ equation), and susceptibility (S) and infectiousness (I) (*p* in the ${{{\mathcal{R}}}}_{0}$ equation). Transmission and infection burden are reduced depending on the effectiveness of these interventions (noted with colors in (**B**)). **B** Changing protective ability of interventions may be due to (i) introducing novel transmission pathways, (ii) effects on intervention effectiveness, or, (iii) disruptions of intervention implementation.

Where *c* is the contact rate, *p* is the probability of transmission between an infectious and susceptible individual, and *γ* is the recovery rate. Interventions aim to control pathogens by reducing contact (*c*), or susceptibility and infectiousness (*p*) (shown conceptually in Fig. 1A). Transmission growth occurs when ${{{\mathcal{R}}}}_{0} > 1$. Thus, interventions aim to reduce ${{{\mathcal{R}}}}_{0}$ below unity.

Because interventions constrain ${{{\mathcal{R}}}}_{0}$, changes in the protection of interventions under global change can lead to increased transmission. Shifts in transmission can be attributable as much to effects on interventions as they are to effects on biological factors, such as vector dispersal patterns, which are more often studied. Global change can modulate the protective effects of these interventions by (i) introducing novel transmission pathways that current treatment programs may not be well-adapted for, (ii) changing the effectiveness of treatments, and (iii) disrupting intervention implementation (Fig. 1B). A key characteristic of global change is its multiplicity of effects. A single global change factor can hinder multiple interventions (Table 1), and a single intervention can be affected by multiple types of global change.

**Table 1:** Ecological suppression of contact, susceptibility, or infectiousness by disease control interventions is affected by global change

	

Each bullet point is an effect of global change, which can be classified as either introducing novel transmission pathways current treatment programs are ill-equipped to handle (*), changing intervention effectiveness (◇), or disrupting interventions (•). Classified according to whether global change challenges the ability of interventions to reduce contact (left), or reduce susceptibility or infectiousness (right).

*Novel transmission pathways* are expected when global change enables transmission in novel environments, or expands transmission beyond the current host-pathogen system (Fig. 1B, (i)). Current treatment programs may not be well-adapted to these unprecedented situations. There are many systems where range expansion of pathogen transmission and disease is anticipated. For example, climate change and land-use change, such as increased agriculture, can increase host availability for tick-borne diseases, promoting wider pathogen host and geographic expansion^15, 16^. As infection burden emerges in novel locations and populations, there may be a delays in supplying proper treatments^5, 16^, especially those with weaker public health infrastructure. Similarly, malaria in Brazil has more recently increased in prevalence among the *garimpo*, i.e., small-scale mining, worker population, who may not have the necessary education, awareness, and resources to prevent infection^17^.

*A change in intervention effectiveness* is expected when current treatments are affected by the environmental factors that may shift with global change (Fig. 1B, (ii)). A key example is the effect of climate change on treatments such as vaccines and antibiotics that normally reduce host susceptibility to infection. The adaptive immune response induced by certain vaccines, e.g., the poliovirus vaccine^18^, depends on climatic factors such as temperature, suggesting there could be complex changes in population susceptibility under changing environmental conditions. In the case of antimicrobial resistance (AMR) during mass drug administration (MDA), increased warming has been associated with higher selection pressures due to increased horizontal gene transfer, where resistance genes can quickly spread throughout microbial populations^19, 20^. Thus, treatment effectiveness can change due to both how hosts and pathogens respond to changes in climate.

*Disruptions to interventions* due to global change can directly affect the ability of interventions to control disease by limiting their deployment (Fig. 1B, (iii)). The most evident examples may be extreme weather events such as cyclones, which can interrupt the deployment of routine interventions (such as bed nets and prophylaxis)^21–23^, and drought or heatwave conditions that disrupt the stability of cold-chains for vaccine delivery^6, 24^. Resistant genes against antibiotics may also be selected due to short-term disruptions^19^. Beyond environmental changes, changes in human mobility in conflict zones^25^, may also hinder access to immunizations and routine care^26^. Especially fragile to logistical disruptions are livestock vaccination programs, as these vaccines must be continuously re-administered for proper protection^27, 28^. In each case, interruptions increase inefficiencies in administration, escalating overall costs in implementation and decreasing program effectiveness. Even short term disruptions can have complex and nonlinear consequences. If a short term disruption in intervention elevates ${{{\mathcal{R}}}}_{0} > 1$, then it may be more difficult to reduce transmission back down below 1 than it was to keep ${{{\mathcal{R}}}}_{0}$ below 1 to begin with, in an ecological phenomenon known as a backwards bifurcation^29^, as is the case for tuberculosis ^30^.

Beyond interventions that directly reduce transmission or disease, surveillance and diagnostic capabilities may also be affected by disruptions, creating gaps in longitudinal datasets with downstream implications for early warning systems^31^. Ultimately, frequent gaps have the potential to strongly reduce data quality and utility, where in the worst case scenario an entire outbreak could go undetected.

## Counteractions that promote robustness of interventions to global change

An array of counteractions are available to mitigate the detrimental effects of global change on infectious disease interventions (Box 1). For example, recent work has suggested that vaccines administered ahead of extreme events could mitigate elevated transmission after climate-driven disruptions^21^, illustrating the potential power of proactive interventions to offset temporarily weakened control programs.

As global change alters the ability of interventions to act as ecological suppressors of transmission, developing theory with ecological models can capture the often nonlinear consequences on pathogen systems^32^. For potential disruptions due to global change, understanding how perturbations are likely to affect the underlying ecology of diseases is important to improve existing interventions. Increased integration of ecological modeling into disease control interventions will be needed to model longer-term spatiotemporal dynamics of global change^33^. Identifying similarities and differences across disease systems experiencing global change may also provide opportunities to develop general theories of optimal counteractions.

Besides a top-down, theory-driven approach, increasingly abundant and diverse data streams can assure that counteractions are empirically informed. For example, advanced remote-sensing surveillance techniques are able to capture observed and environmental variables in high resolution^34^. Detailed mobility data can document changes in population-dynamics, informing how interventions are likely to be impacted by changes at local scales—the first step towards determining how we might adjust^35, 36^. The vulnerability of current diagnostics capabilities to global change (Table 1) will necessitate the development of additional strategies to improve targeting of interventions. Rapid diagnostic testing kits may be more efficient than standard microscopy techniques for case identification, and can be incorporated into test and treat programs^37, 38^. Additionally, local-scale surveillance has been shown to improved trust in intervention infrastructure[8], and further investments are especially important in the global south, as well as other areas most acutely affected by global change.

Box 1: Types of counteractions against the effects of global change on public health interventions [1.] Development of the knowledge base of anticipated consequences of global change on public health interventions using both theory- and data-driven approaches

[2.] Novel intervention technologies with increased effectiveness, longer effectiveness duration, or more robust administration systems that can overcome challenges induced by global change

[3.] Diagnostic tests and innovative testing strategies to improve targeting of response campaigns after interventions are disrupted by global change

## Conclusion

The success of established disease control interventions has led several infectious diseases to the brink of local or regional elimination; global change and disease re-emergence threaten to undermine this immense progress^39^. Current global agendas have ambitious goals for burden reduction and regional elimination of many diseases, including malaria, HIV, and some neglected tropical diseases^34, 40, 41^. Reaching these goals will depend on our ability to work across disciplines and counteract the challenges global change pose on interventions. To do so, we must build a strong theoretical foundation that allows us to anticipate changes in pathogen control, invest in data infrastructure to monitor progress, and continue to build local capacity for disease control.

## Data Availability

No datasets were generated or analysed during the current study.

## References

[1] Baker, R. E. et al. Infectious disease in an era of global change. *Nat. Rev. Microbiol.* **20**, 193–205 (2022). 10.1038/s41579-021-00639-z PMC8513385 34646006

[2] Carlson, C. J. et al. Pathogens and planetary change. *Nat. Rev. Biodivers.* **1**, 32–49 (2025). 10.1038/s44358-024-00005-w PMC7618822 41789152

[3] Childs, M. L., Nova, N., Colvin, J. & Mordecai, E. A. Mosquito and primate ecology predict human risk of yellow fever virus spillover in Brazil. *Philos. Trans. R. Soc. B* **374**, 20180335 (2019). 10.1098/rstb.2018.0335 PMC6711306 31401964

[4] Ryan, S. J., Lippi, C. A. & Zermoglio, F. Shifting transmission risk for malaria in Africa with climate change: a framework for planning and intervention. *Malar. J.* **19**, 1–14 (2020). 10.1186/s12936-020-03224-6 PMC7193356 32357890

[5] Mordecai, E. A., Ryan, S. J., Caldwell, J. M., Shah, M. M. & LaBeaud, A. D. Climate change could shift disease burden from malaria to arboviruses in Africa. *Lancet Planet. Health* **4**, e416–e423 (2020). 10.1016/S2542-5196(20)30178-9 PMC7490804 32918887

[6] Mahmud, A. S., Martinez, P. P., He, J. & Baker, R. E. The impact of climate change on vaccine-preventable diseases: insights from current research and new directions. *Curr. Environ. Health Rep.* **7**, 384–391 (2020). 10.1007/s40572-020-00293-2 PMC7585557 33099754

[7] Oakes, R. et al. A future agenda for research on climate change and human mobility. *International Migration* (2023).

[8] Worsley-Tonks, K. E. et al. A framework for managing infectious diseases in rural areas in low-and middle-income countries in the face of climate change–East Africa as a case study. *PLOS Glob. Public Health* **5**, e0003892 (2025). 10.1371/journal.pgph.0003892 PMC11781624 39883787

[9] Ebi, K. L., Kovats, R. S. & Menne, B. An approach for assessing human health vulnerability and public health interventions to adapt to climate change. *Environ. Health Perspect.* **114**, 1930–1934 (2006). 10.1289/ehp.8430 PMC1764166 17185287

[10] Watts, N. et al. Health and climate change: policy responses to protect public health. *Lancet* **386**, 1861–1914 (2015). 10.1016/S0140-6736(15)60854-6 26111439

[11] Madon, T., Hofman, K. J., Kupfer, L. & Glass, R. I. Implementation science (2007). 10.1126/science.1150009 18079386

[12] Johnson, P. T., De Roode, J. C. & Fenton, A. Why infectious disease research needs community ecology. *Science* **349**, 1259504 (2015). 10.1126/science.1259504 PMC4863701 26339035

[13] Drake, J. Why ecologists study infectious diseases and what we can learn about public health by studying nature. *Forbes* (2020).

[14] Van den Driessche, P. & Watmough, J. Reproduction numbers and sub-threshold endemic equilibria for compartmental models of disease transmission. *Math. Biosci.* **180**, 29–48 (2002). 10.1016/s0025-5564(02)00108-6 12387915

[15] Burrows, H. et al. A multi-year assessment of blacklegged tick (Ixodes scapularis) population establishment and Lyme disease risk areas in Ottawa, Canada, 2017-2019. *PLoS One* **16**, e0246484 (2021). 10.1371/journal.pone.0246484 PMC7861446 33539458

[16] Shahzad, S. et al. Ticks and tickborne diseases in Global South countries: Impact and implications of environmental changes. *Front. Tropical Dis.* **6**, 1597236 (2025).

[17] Murta, F. L. et al. Perceptions about malaria among Brazilian gold miners in an Amazonian border area: perspectives for malaria elimination strategies. *Malar. J.* **20**, 286 (2021). 10.1186/s12936-021-03820-0 PMC8236171 34174880

[18] Zhang, K. et al. Impact of climate change on vaccine responses and inequity. *Nat. Clim. Change* **14**, 1216–1218 (2024). 10.1038/s41558-024-02192-y PMC12021441 40276125

[19] Ni, Y., Zhao, J., Yuan, Y. & Feng, B. Climate change and antimicrobial resistance: a global-scale analysis. *BMC Infect. Dis.* **25**, 1–15 (2025). 10.1186/s12879-025-11616-9 PMC12482477 41023856

[20] Li, W. et al. Changing climate and socioeconomic factors contribute to global antimicrobial resistance. *Nat. Med.* **31**, 1798–1808 (2025). 10.1038/s41591-025-03629-3 40295742

[21] Rice, B. L. et al. Vaccination to mitigate climate-driven disruptions to malaria control in Madagascar. *Science* **389**, eadp5365 (2025). 10.1126/science.adp5365 40674494

[22] Searle, K. M. et al. Household storm damage limits use of and access to insecticide treated bednets in Mozambique. *Ann. Hum. Biol.* **52**, 2461145 (2025). 10.1080/03014460.2025.2461145 40047173

[23] Symons, T. L. et al. Projected impacts of climate change on malaria in Africa. *Nature* **651**, 390–396 (2026). 10.1038/s41586-025-10015-z PMC12979204 41606340

[24] Caldwell, J. M. et al. Climate predicts geographic and temporal variation in mosquito-borne disease dynamics on two continents. *Nat. Commun.* **12**, 1233 (2021). 10.1038/s41467-021-21496-7 PMC7902664 33623008

[25] Marou, V. et al. The impact of conflict on infectious disease: a systematic literature review. *Confl. Health* **18**, 27 (2024). 10.1186/s13031-023-00568-z PMC11000310 38584269

[26] Mbaeyi, C. Polio vaccination activities in conflict-affected areas. *Hum. Vaccines Immunotherapeutics* **19**, 2237390 (2023). 10.1080/21645515.2023.2237390 PMC10392756 37489591

[27] Annapragada, A. et al. Modeling the impact of Newcastle disease virus vaccinations on chicken production systems in northeastern Madagascar. *Front. Vet. Sci.* **6**, 305 (2019). 10.3389/fvets.2019.00305 PMC6775217 31612142

[28] Liu, C.-C., Chow, Y.-H., Chong, P. & Klein, M. Prospect and challenges for the development of multivalent vaccines against hand, foot and mouth diseases. *Vaccine* **32**, 6177–6182 (2014). 10.1016/j.vaccine.2014.08.064 25218294

[29] Hadeler, K. P. & Van den Driessche, P. Backward bifurcation in epidemic control. *Math. Biosci.* **146**, 15–35 (1997). 10.1016/S0025-5564(97)00027-8 9357292

[30] Wangari, I. M. & Stone, L. Backward bifurcation and hysteresis in models of recurrent tuberculosis. *PLoS ONE* **13**, e0194256 (2018). 10.1371/journal.pone.0194256 PMC5863985 29566101

[31] Neta, G. et al. Advancing climate change health adaptation through implementation science. *Lancet Planet. Health* **6**, e909–e918 (2022). 10.1016/S2542-5196(22)00199-1 PMC9669460 36370729

[32] Richard, L., Gauvin, L. & Raine, K. Ecological models revisited: their uses and evolution in health promotion over two decades. *Annu. Rev. Public Health* **32**, 307–326 (2011). 10.1146/annurev-publhealth-031210-101141 21219155

[33] Metcalf, C. J. E., Grenfell, B. T. & Graham, A. L. Disentangling the dynamical underpinnings of differences in sars-cov-2 pathology using within-host ecological models. *PLoS Pathog.* **16**, e1009105 (2020). 10.1371/journal.ppat.1009105 PMC7732095 33306746

[34] Arisco, N. J., Peterka, C., Diniz, C., Singer, B. H. & Castro, M. C. Ecological change increases malaria risk in the brazilian amazon. *Proc. Natl. Acad. Sci. USA.* **121**, e2409583121 (2024). 10.1073/pnas.2409583121 PMC11536126 39432796

[35] Wesolowski, A., Buckee, C. O., Engø-Monsen, K. & Metcalf, C. J. E. Connecting mobility to infectious diseases: the promise and limits of mobile phone data. *J. Infect. Dis.* **214**, S414–S420 (2016). 10.1093/infdis/jiw273 PMC5144902 28830104

[36] Souza Jr, C. M. et al. Reconstructing three decades of land use and land cover changes in Brazilian biomes with Landsat archive and Earth Engine. *Remote Sens.* **12**, 2735 (2020).

[37] Rachlin, A. et al. Use of measles and rubella rapid diagnostic tests to improve case detection and targeting of vaccinations. *Vaccines* **12**, 823 (2024). 10.3390/vaccines12080823 PMC11360261 39203949

[38] Oyeyemi, O. T., Ogundahunsi, O., Schunk, M., Fatem, R. G. & Shollenberger, L. M. Neglected tropical disease (NTD) diagnostics: current development and operations to advance control. *Pathog. Glob. Health* **118**, 1–24 (2024). 10.1080/20477724.2023.2272095 PMC10769148 37872790

[39] Mackey, T. K. et al. Emerging and reemerging neglected tropical diseases: a review of key characteristics, risk factors, and the policy and innovation environment. *Clin. Microbiol. Rev.* **27**, 949–979 (2014). 10.1128/CMR.00045-14 PMC4187634 25278579

[40] Bangert, M., Molyneux, D. H., Lindsay, S. W., Fitzpatrick, C. & Engels, D. The cross-cutting contribution of the end of neglected tropical diseases to the sustainable development goals. *Infect. Dis. Poverty* **6**, 1–20 (2017). 10.1186/s40249-017-0288-0 PMC5379574 28372566

[41] Matola, B. W., Mapahla, L. & Nyasulu, J. C. Y. Malawi’s progress towards UNAIDS 95-95-95 fast-track targets: Who is lagging? *Afr. J. AIDS Res.* 1–7 (2025). 10.2989/16085906.2025.2477090 40366778

